# Molecular Dynamics Simulation of Interfacial Effects in PBT-Based Azide Propellants Under Tensile Deformation

**DOI:** 10.3390/polym17070885

**Published:** 2025-03-26

**Authors:** Hongjun Liao, Jiangyan Lv, Peng Cao, Liang Cao, Renlong Huang, Xianqiong Tang

**Affiliations:** 1 School of Mechanical Engineering and Mechanics, Xiangtan University, Xiangtan 411105, China; 202221572238@smail.xtu.edu.cn (H.L.); 202221572230@smail.xtu.edu.cn (R.H.); 2 The 41st Institute of Fourth Academy of China Aerospace Science and Technology Corporation, Xi’an 710025, China; huiweimail@163.com; 3 College of Architecture and Civil Engineering, Beijing University of Technology, Beijing 100124, China; caopeng@bjut.edu.cn (P.C.); liangcao988@emails.bjut.edu.cn (L.C.)

**Keywords:** molecular dynamics simulation, interfacial effect, uniaxial tensile deformation, AP crystal defects, crosslinking degree

## Abstract

The mechanical properties of PBT-based azide propellants, composed of a 3,3′-bis(azidomethyl)oxetane/tetrahydrofuran (PBT) copolymer matrix and defective ammonium perchlorate (AP) crystals, are significantly influenced by the matrix–crystal interface. This study employed molecular dynamics (MD) simulations to examine interfacial effects on mechanical performance under uniaxial tensile deformation. Models with varying cross-linking densities (70%, 80%, 90%) and AP defect widths (20 Å, 30 Å, 40 Å) were analyzed to assess the effects of temperature, strain rate, cross-linking degree, and defect size on interfacial adhesion strength and failure mechanisms. Results indicate that at low temperatures, the interface exhibited high stress peaks and brittleness characteristics, transitioning to plastic flow and enhanced ductility at higher temperatures. Cross-linking density significantly affects interfacial strength: a 90% cross-linking degree achieved the highest stress peak and optimal tensile resistance, whereas lower cross-linking resulted in weaker stress transfer and accelerated post-peak stress decay. Higher strain rates increased peak stress and shortened deformation times, while lower strain rates promoted molecular rearrangement, enhancing tensile resistance. Defect size also plays a crucial role, with smaller defects maintaining interfacial dominance, whereas larger defects shift failure toward the bulk matrix, reducing stress transfer efficiency. These findings provide atomic-scale insights into interfacial defects and key material parameters, offering theoretical guidance for optimizing the structural stability of composite propellants.

## 1. Introduction

Solid propellants, a class of fuels characterized by storage stability and ready-to-use properties, are widely employed in military missiles and small launch vehicles [1,2,3,4,5,6]. Among these, azide polyether propellants—notably PBT-based azide composite propel-lants—are high-energy formulations extensively used in spacecraft and missiles. These propellants are synthesized by crosslinking 3,3-bis(azidomethyl)oxetane (BAMO) and tetrahydrofuran (THF) random copolyether (PBT) with 2,6-toluene diisocyanate (TDI), tri-methylolpropane (TMP), and triethylene glycol (TEG) to form a three-dimensional net-work structure [7,8].

In PBT-based azide composite propellants, the combined mass of the PBT crosslinked binder and ammonium perchlorate (AP) solid filler accounts for over 80% of the total composition. Consequently, interfacial debonding between the PBT matrix and AP filler poses a critical challenge during transportation and combustion of propellants, where vibrational and accelerative forces induce tensile stresses that weaken adhesion [9,10,11,12,13,14]. Such debonding can lead to uneven combustion or structural failure, compromising propellant performance. Enhancing interfacial adhesion through material optimization is therefore essential to ensure mechanical stability.

In recent years, numerous studies have been conducted to investigate interfacial interaction in propellant [15,16,17,18,19,20,21,22,23]. For instance, Wu et al. [16] investigated the interfacial failure mechanisms of HTPB propellant at low strain rates through experiments and finite element modeling, demonstrating that tensile strength of propellant increases with strain rate. Zou et al. [19] developed a cohesive model that effectively captures mechanical behavior of particle/matrix interfaces in composite solid propellants, critical for the overall mechanical properties of propellants. Yeager et al. [21] demonstrated via nanoindentation that HMX–Estane interfaces with nitrated plasticizers exhibit reduced strength compared to those without. Computational approaches, such as the normalized crack length parameter proposed by Lei et al. [22], have enabled finite element modeling of thermomechanical responses in propellant structures. Gu et al. [23] further correlated the meso-structural state with the macro-mechanical properties through experimental and simulation methods, providing insights for predicting the macro-mechanical properties and failure mechanisms of composite propellants.

Although previous studies have provided a great deal of valuable information for understanding interfacial behavior, direct observation of nanoscale interfacial features (e.g., elongation deformation of molecular chain, defect distribution, and interfacial thickness) is still challenging due to the limitations of current high-resolution measurement techniques. Molecular dynamics (MD) simulations can overcome these obstacles and provide insight into the structural and dynamic behavior at the atomic level. MD allows precise control of parameters (e.g., temperature, strain rate) and systematic analysis of material response under extreme conditions. This makes MD a key technique for studying the relationship between the microstructure and the macroscopic mechanical properties of materials [24,25,26,27,28,29,30]. For example, Lv et al. [29] used MD to investigate the fracture mechanism of the TATB-F2314 interface with temperature and strain rate changes, revealing the effects of temperature and strain rate on the mechanical properties of the interface. Dong et al. [30] investigated the interfacial interaction between AP and HTPB in solid propellants through all-atom molecular dynamics simulations, finding that the matrix structure, tensile rate, and contact area had a significant effect on the mechanical properties, which provided theoretical support for propellant design. Similarly, MD simulations can elucidate the effects of interfacial defects (e.g., voids, “insufficient crosslinking”) on the mechanical properties of the PBT/AP system, which can provide important guidance for defect mitigation strategies [31,32,33,34,35,36,37]. “Insufficient crosslinking” means that a portion of the cross-linking points do not react. If the cross-linking points react, then new bonds are created, improving the mechanical properties of PBT. If the cross-linking points do not react, then there is a potential weak spot near the cross-linking points. Li et al. [35] revealed that void defects in solid propellant generate hot spots under shock, which affects the detonation and mechanical performance. Jia et al. [37] utilized all-atom MD simulations to investigate the interfacial adhesion mechanisms between PBT azide propellant matrices and defective AP fillers, revealing that increased matrix crosslinking and optimal defect widths (30 Å) enhance interfacial adsorption strength, while temperature variations induce dynamic fluctuations in binding energy and interfacial structures.

In previous studies, the effects of insufficient crosslinking, tensile rate, temperature, and AP defects on the mechanical properties of the PBT matrix–AP interface have not been systematically considered. In this study, the PBT matrix–defect AP interface with insufficient crosslinking under uniaxial tensile loading was investigated using MD simulation, and the effects of tensile rate, temperature, and defect size on the interfacial properties were analyzed. Based on the outcomes, this study aims to provide atomic-scale insights into interfacial defects and offer theoretical guidance for optimizing the design of composite propellants. The work is structured as follows: Section 2 details the construction of the PBT cross-linked matrix, AP crystal models, and simulation parameters. Section 3 discusses stress-displacement relationships under varying tensile conditions, and Section 4 summarizes key conclusions.

## 2. Materials and Methods

Molecular models were constructed using BIOVIA Materials Studio 2019 software. Interfacial relaxation simulations were conducted in the Forcite module, while crosslinking reactions and tensile processes were executed through Perl scripts. All simulations utilized the COMPASS II force field, which is specifically designed for MD simulations and provides robust support for polymeric materials. The force field parameters of COMPASS II are obtained based on the fitting results of quantum chemistry and also corrected based on the experimental results [38,39,40]. It has been successfully applied in the study of explosives and propellants [41,42,43,44,45,46].

### 2.1. Initial Model

**Figure 1:**
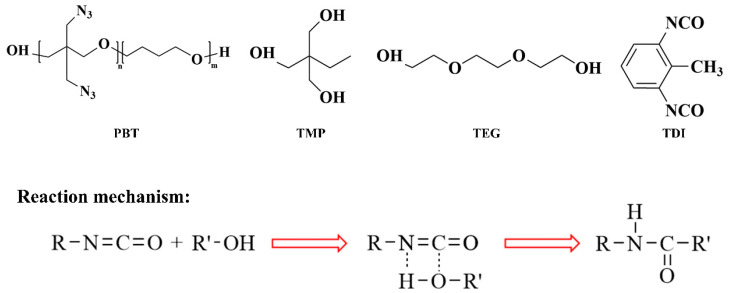
Crosslinking reaction mechanism.

Figure 1 illustrates the molecular structures of four key components in the PBT-based azide composite propellant system: binder polymer PBT, curing agent TDI, cross-linking agent TMP, and chain extender TEG. The schematic diagram further demonstrates the crosslinking reaction mechanism governing the formation of this energetic material system [37]. Hydroxyl groups on PBT, TMP, and TEG react with isocyanate groups on TDI to form urethane linkages. TMP, with its trifunctional nature, acts as the crosslinking agent, creating a branched network structure, while the bifunctional TEG serves as a chain extender.

To ensure complete reaction between the [-NCO] and [-OH] groups, a 1:1 molar ratio was employed in the crosslinking system. Here, [-R] represents the hydrocarbon general formula in TDI, and [-R’] denotes the hydrocarbon or alkyl general formula in PBT, TMP, and TEG. The molecular ratio of the components in the crosslinking reaction formulation was PBT:TDI:TMP:TEG = 55:100:5:35. Based on this ratio, molecular models were placed in a low-density 3D periodic cubic box with an initial density of 0.3 g/cm^3^. Global optimization was performed to eliminate high-energy regions before conducting molecular dynamics (MD) simulations using the COMPASS II force field and the isothermal-isobaric (NPT) ensemble. Global optimization refers to the elimination of unreasonable molecular configurations through energy minimization, annealing, and other methods. The simulations ran for 3 ns with an integration time step of 1 fs. The energy-minimized structure was used to construct a system with a density of 1.15 g/cm^3^, serving as the initial configuration for crosslinking simulations.

In this study, a probabilistic bonding method controlled by the reaction radius was utilized to achieve a precise regulation of the crosslinking degree by adjusting the cross-linking reaction radius, where the crosslinking degree is the ratio of the reacted groups to all reactive groups. The C atom in the [-NCO] group and the O atom in the [-OH] group were designated as reactive groups. When the distance between C atom in the [-NCO] group and the O atom in the [-OH] group was less than the target crosslinking radius, the reaction probability was set to 50%. As the crosslinking reaction progressed, the number of bonds formed gradually increased, leading to an increase in the crosslinking degree. The crosslinking process was terminated when the crosslinking degree reached the preset threshold. Cross-linked molecular models with degrees of 90%, 80%, and 70% were recorded. After a cross-linking degree of 90%, the uncross-linked atoms usually have large spatial distances, and a long time is required for their reaction. In order to save calculation costs, it is therefore limited to 90%.

The AP crystal parameters were derived from single-crystal neutron diffraction data [47]. The AP crystal exhibits an orthorhombic structure with the space group Pnma (62), unit cell parameters of a = 8.94 Å, b = 5.89 Å, c = 7.3 Å, and α = β = γ = 90°. The crystal density is 1.95 g/cm^3^. The (1 0 1) crystal plane, which has a high degree of exposure, was selected for study. A repeating unit with a depth of 13 Å was cut along the (1 0 1) plane, and a supercell periodic structure of (13 × 7 × 13) was constructed for simulation and analysis.

### 2.2. Establishment of the PBT-AP Interface Model

PBT-based azide propellant is a multiphase heterogeneous material. During preparation, manufacturing limitations can introduce initial defects, including (1) voids and bubbles due to air entrapment in the binder matrix under non-vacuum conditions; (2) particle fragmentation from collisions between solid filler particles during mixing; and (3) interfacial defects caused by inconsistent deformation due to differences in thermal conductivity during curing and cooling. These defects reduce structural strength and impair mechanical properties [48,49].

In this study, idealized rectangular particle fragmentation defects were used to represent potential interfacial defects. Rectangular defects with a depth of 40 Å and widths of 20 Å, 30 Å, and 40 Å were excavated in the AP layer. After constructing the PBT matrix–AP interface system, atomic coordinates were relaxed using the conjugate gradient method, with a force convergence tolerance of 10^−8^ kcal/mol/Å. A 60 Å vacuum layer was introduced at the top of the crosslinked layer to prevent interactions between the crosslinked layer and periodic images of the AP layer during stretching [50,51]. MD simulations were performed under periodic boundary conditions using the NVT ensemble, with a simulation temperature of 500 K, an integration time step of 0.2 fs, and a total duration of 8 ns. The Anderson thermostat maintained system temperature, and the Particle-Particle-Particle-Mesh (PPPM) method handled Coulomb interactions.

**Figure 2:**
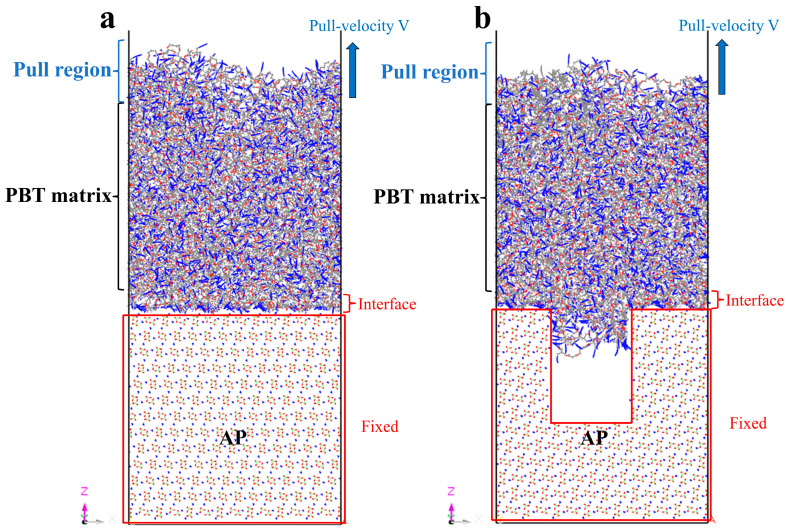
Schematic of the uniaxial stretching process for (**a**) defect-free interface and (**b**) defective interface models.

Two models were constructed: (1) a two-phase adsorption model with PBT matrices of varying crosslinking densities placed above a pristine AP surface (Figure 2a) and (2) a two-phase defect adsorption model with the PBT matrix placed above an AP surface containing rectangular defects (Figure 2b).

### 2.3. Uniaxial Tension Simulation

Dynamic uniaxial tensile simulations were performed on the PBT matrix–AP interface structure to investigate the effects of temperature and strain rate on interfacial mechanical properties. Figure 2 illustrates the uniaxial tensile process, where a constant rate along the z-axis was applied to the tensile region. The simulation consisted of stretching and equilibration phases. Tensile deformation along the z-axis was applied with a time step of 0.5 fs during the dynamic stretching phase, using the NVE ensemble. The AP crystal was fixed, and the topmost portion of the PBT matrix (approximately 20 μm) was treated as a rigid body, displaced along the z-axis at a prescribed velocity. The remaining PBT matrix was allowed to deform in response. After stretching, equilibration simulations were performed by removing the horizontal stretching along the z-axis, followed by thermal equilibrium relaxation for 1 ns to ensure system stability. Interfacial adhesion stress was calculated by dividing the traction force applied to the rigid portion of the PBT matrix by the cross-sectional contact area. Average stress values for each relaxation step were computed, and a stress–displacement curve was plotted to describe interfacial deformation characteristics.

## 3. Results and Discussion

### 3.1. Determination of Interface Area

In the PBT matrix–AP interface model, the portion of the PBT matrix in contact with the AP surface exhibited distinct properties compared to the bulk PBT matrix. After an equilibrium process under the NVT ensemble, the model was divided into 500 equally thick slices along the z-axis, each with a thickness of 1.04 Å. The atomic mass within each slice was calculated, and the density of each slice was determined by calculating the mass of the atom and the volume of the slice. Density fluctuations in each slice were analyzed to identify the mixed phase, with the width of the fluctuation region defined as the mixed phase thickness. To minimize errors, the last five frames from the MD equilibration were extracted, and the mixed phase thickness was statistically averaged [52].

**Figure 3:**
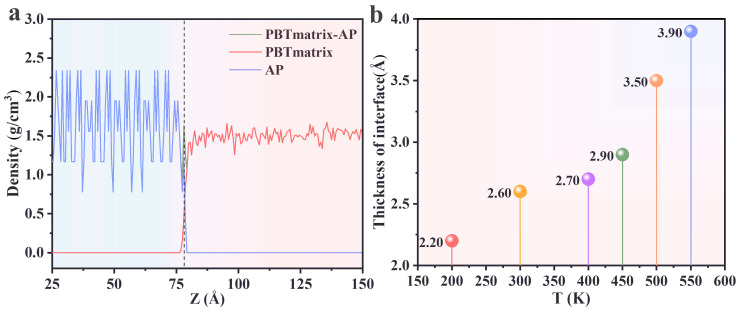
(**a**) Density variation along the z-direction of the AP, PBT matrix, and PBT–AP interface at T = 300 K. (**b**) Thickness of the interfacial region at different temperatures.

Figure 3a illustrates the density variation of the PBT matrix along the z-axis at 298 K. A significant density difference between the AP and PBT matrix was observed near the interface. The density curves of the AP and PBT matrix regions began with a large fluctuation zone, and as they approached the interface, their densities decreased. The intersection boundary of these curves defined the interfacial region thickness. The effect of temperature on the interfacial region thickness was investigated, as shown in Figure 3b. The thickness of the interfacial region was minimally affected by temperature, with only a slight increase observed as temperature rose.

### 3.2. Effect of Temperature

**Figure 4:**
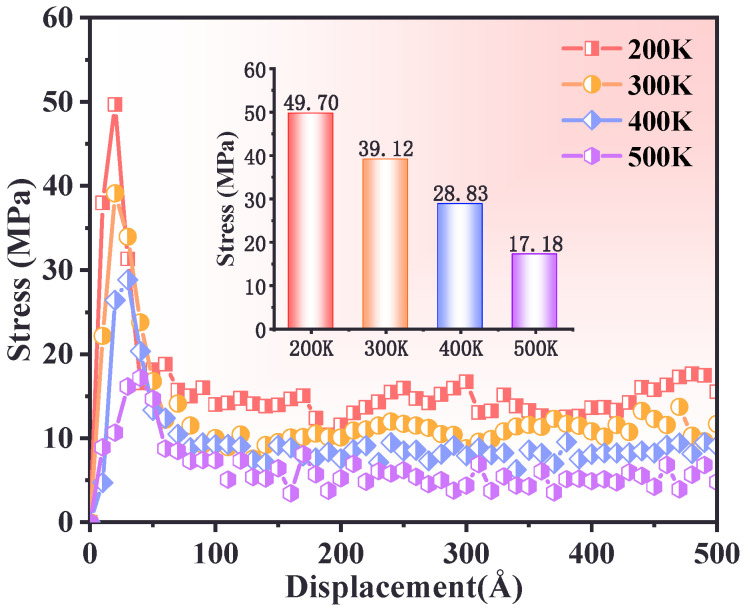
Relationship between interfacial stress–displacement curves at different temperatures.

In order to further understand the effect of temperature on the tensile mechanical properties of interfaces, the quasi-static tensile experiments were conducted at temperatures of 200 K, 300 K, 400 K, and 500 K, with a strain rate of 1 × 10^10^ s^−1^. The stress–displacement curves at different temperatures are shown in Figure 4. At 200 K, the material exhibited a higher peak stress during the tensile process. As the tensile distance increased, stress rose sharply and reached its maximum within a short distance. This suggests that at low temperatures, molecular chain mobility is limited, leading to elongation of polymer chains with a sharp decline in stress after reaching the peak. At 300 K and 400 K, the peak stress gradually decreased with increasing temperature, and the peak occurrence shifted to the right. The increased molecular chain mobility at higher temperatures facilitated slippage, reducing peak stress and resulting in a more gradual elastic-to-plastic transition. At 500 K, the stress peak significantly decreased, and the stress curve exhibited a flatter profile with a slower decline. This indicates enhanced molecular chain fluidity, resulting in increased interfacial deformation capability and pronounced viscoelasticity and ductility at elevated temperatures.

### 3.3. Effect of Crosslinking Degree of PBT Matrix

**Figure 5:**
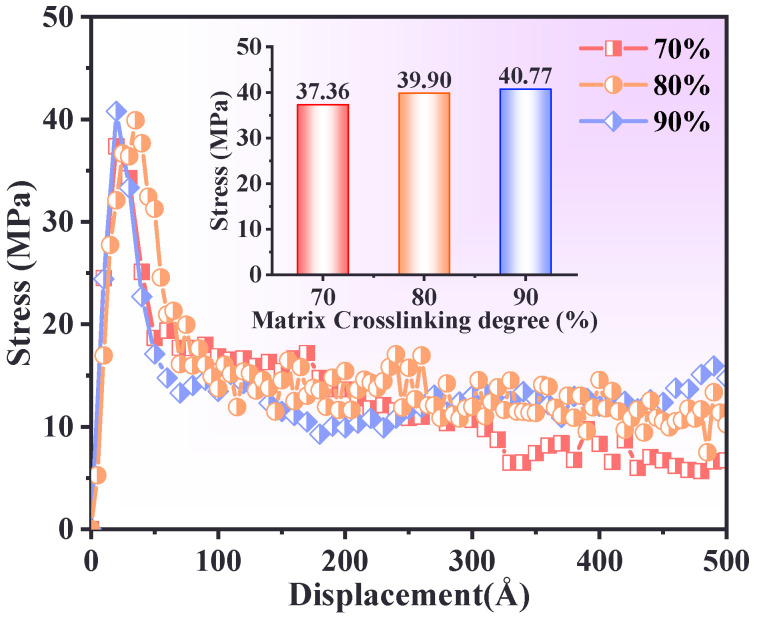
Interfacial stress–displacement curve relationships for different matrix crosslinking degrees.

The effect of crosslinking degree on interfacial mechanical properties was investigated by calculating stress–displacement relationships for PBT matrices with 70%, 80%, and 90% crosslinking degrees at 300 K and a tensile rate of 1 × 10^10^ s^−1^. The results are shown in Figure 5. For all crosslinking degrees, the initial tensile stress increased rapidly with tensile distance until reaching a peak stress value. During this phase, the material primarily exhibited elastic deformation. All curves stabilized at a tensile distance of 200–300 Å, indicating a stress saturation stage with minimal further changes. Beyond 300–500 Å, the stress for the 70% crosslinked matrix decreased significantly due to its looser network structure, which led to partial molecular chain separation. The 90% crosslinked matrix exhibited the highest tensile strength due to stronger intermolecular interactions requiring greater external force for deformation. In contrast, the 70% crosslinked matrix showed a rapid decline in stress after peaking, attributed to reduced crosslinking points and increased molecular chain slippage. The 80% crosslinked matrix exhibited an intermediate response, with a delayed peak stress compared to the 90% crosslinked sample, likely due to reduced stress transfer efficiency.

### 3.4. Effect of Strain Rate

**Figure 6:**
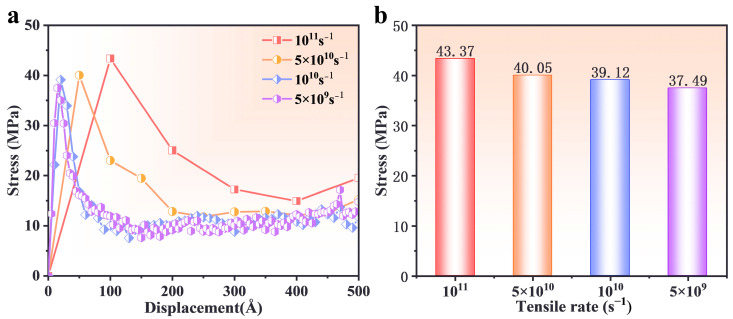
(**a**) Interfacial stress–displacement curve relationships at different tensile rates. (**b**) Maximum stress at different tensile rates.

Dynamic uniaxial tensile simulations were conducted at strain rates of 1 × 10^11^, 5 × 10^10^, 1 × 10^10^, and 5 × 10^9^ s^−1^ at 300 K. Due to computational constraints, the simulation box size was on the nanometer scale, leading to higher stresses in MD simulations [53]. Figure 6 shows the stress–displacement curves for different strain rates. Initially, all curves exhibited a linear increase, corresponding to elastic deformation. After reaching peak stress, further PBT matrix dissociation caused a rapid decline in stress. Higher strain rates resulted in increased peak stress due to restricted molecular segment motion, reducing relaxation time and delaying the transition to the plastic phase. Stress oscillations observed in all curves can be attributed to thermal fluctuations.

**Figure 7:**
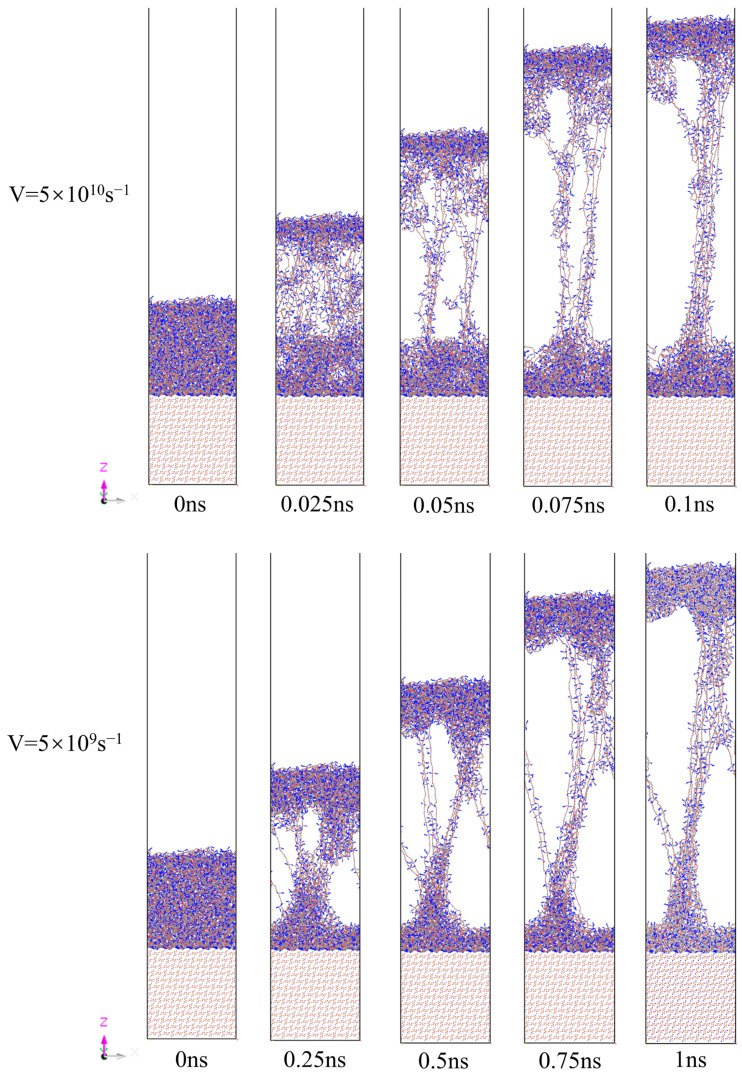
Snapshots of the AP–PBT matrix interface model at different stretching rates. Color scheme: cyan (carbon), blue (nitrogen), red (oxygen).

Snapshots of the uniaxial tensile deformation process at different rates are shown in Figure 7. To investigate the effect of strain rate on the stress distribution in the PBT matrix–AP interface model, simulation snapshots were extracted for strain rates of 5 × 10^10^ s^−1^ and 5 × 10^9^ s^−1^. During the interfacial tensile process at these two different rates, the system maintained its integrity. Notably, the interfacial region exhibited significantly higher resistance to tensile deformation compared to the non-interfacial region (i.e., the bulk PBT matrix region), with molecular chain sliding behavior occurring exclusively in the non-interfacial region. This phenomenon indicates that the interfacial interactions between AP and the PBT matrix play a critical role in stress transfer and dispersion during the tensile process. In the stress response of the PBT matrix molecules, due to the bonding and non-bonding interactions between atoms, the PBT matrix molecules remained connected through a fibrous structure without slippage, fully demonstrating the excellent plasticity of the cross-linked polymeric PBT material.

It can be observed that there was no complete separation of the matrix at the interface. The tensile results indicate that AP exhibited strong adsorption affinity to PBT, and the deformation primarily occurred within the PBT matrix during the stretching process. Under the two strain rates of 5 × 10^10^ s^−1^ and 5 × 10^9^ s^−1^, the PBT matrix molecules exhibited significant differences in strain patterns. At the higher strain rate (0.025 ns), the separation of the main body of the PBT matrix molecules was slower and incomplete, with a thicker bulk PBT matrix region remaining near the interfacial area. In contrast, at the lower strain rate (0.25 ns), elongation of molecular chains had already occurred significantly, resulting in a thinner bulk PBT matrix region near the interfacial area. This difference can be explained by the dynamic response mechanism of molecular chains: under high-rate external forces, the rearrangement and slippage of molecular chains are time-constrained, leading to uneven stress distribution, whereas under low-rate external forces, molecular chains have sufficient time to rearrange, enabling uniform deformation.

Furthermore, morphological analysis revealed the significant influence of strain rate on the motion behavior of molecular chains. After high-rate tensile deformation (0.1 ns), the bulk PBT matrix region exhibited a rope-like structure, whereas after low-rate tensile deformation (1 ns), it displayed a hollow structure extending from the inside outward. This morphological difference further confirms the significant impact of external force loading rate on the motion behavior of molecular chains. Under high-rate conditions, the cooperative motion of molecular chains is restricted, while under low-rate conditions, molecular chains can achieve more extensive slippage and separation.

### 3.5. Effect of AP Defect

To investigate the effect of interfacial defects, stress–displacement relationships were analyzed for PBT matrix–AP interfaces with defect widths of 20 Å, 30 Å, and 40 Å under 90% crosslinking, 300 K, and a tensile rate of 1 × 10^10^ s^−1^.

**Figure 8:**
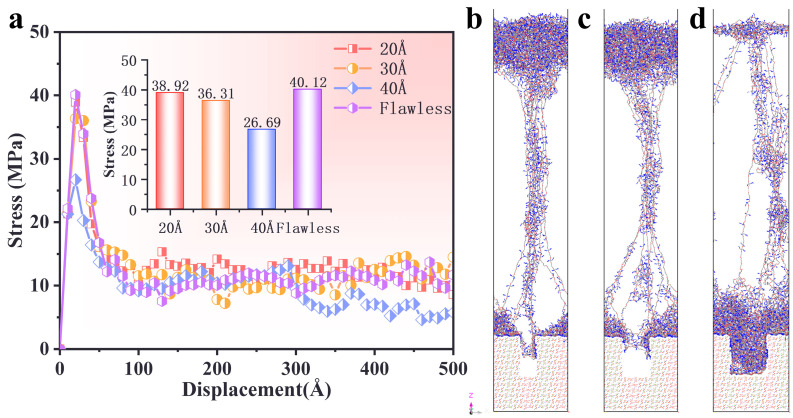
(**a**) Stress–displacement curve relationships at interfaces with different matrix defect widths. (**b**–**d**) Snapshots of uniaxial tensile deformation results at different defect interfaces. Color scheme: cyan (carbon), blue (nitrogen), red (oxygen).

The stress–displacement curves for interfaces with varying defect widths are shown in Figure 8a. It can be observed that the stress peaks for the defect-free interface and those with defect widths of 20 Å, 30 Å, and 40 Å were 40.115 MPa, 38.92 MPa, 36.314 MPa, and 26.687 MPa, respectively. At a defect width of 20 Å, the stress of the material increased rapidly and reached a relatively high peak, which was close to that of the defect-free interface. This indicates that a smaller AP defect width has a minor impact on the interfacial mechanical properties. At a defect width of 30 Å, the stress peak slightly decreased as the defect width increased. This suggests that the stress-bearing capacity of the material declines with larger defect widths, but it still maintains relatively good mechanical performance. At a defect width of 40 Å, the stress peak was the lowest and decreased rapidly. This indicates that when the defect width reaches 40 Å, the tensile strength of the material significantly decreases, and the interfacial defect width severely compromises the mechanical performance of the interface. Additionally, at a tensile distance of 300–500 Å, the stress levels during the plastic deformation phase for interfaces with defect widths of 20 Å and 30 Å were essentially comparable to that of the defect-free interface. The stress level for the interface with a 40 Å defect width was significantly lower, demonstrating that larger defects of 40 Å noticeably weaken the interfacial mechanical performance.

To further explore the impact of interfacial defects on the tensile results, snapshots of the uniaxial tensile deformation outcomes for different defective interfaces are shown in Figure 8b–d. In the interface models with three different defect widths (20 Å, 30 Å, and 40 Å), the uniaxial tensile experimental results revealed that, despite the varying defect widths, none of the models experienced complete disconnection. The PBT matrix molecules remained connected through a fibrous structure. This phenomenon indicates that even in the presence of interfacial defects, the PBT matrix molecules can maintain structural continuity through intermolecular bonding and non-bonding interactions, demonstrating the toughness of polymeric materials in the interfacial region.

Specifically, in the models with defect widths of 20 Å and 30 Å, the tensile behavior exhibited similar characteristics: the bulk structure of the PBT matrix was primarily stretched away from the interfacial region, with the molecular chain sliding behavior concentrated near the interfacial area. The fibrous structure near the interfacial region showed a contracted middle section and a divergent connection centered around the defect at the interfacial end. This strain behavior suggests that, under smaller defect widths, the stress distribution in the interfacial region is relatively uniform, and the influence of the defect on the overall mechanical behavior is limited. The PBT matrix molecules primarily adapt to external forces through the slippage and rearrangement of molecular chains. However, in the model with a defect width of 40 Å, the tensile behavior changed significantly. The PBT matrix was filled into the defect, and the bulk structure adsorbed toward the interfacial region, with the molecular sliding behavior shifting to the near-tensile area. The fiber structure of the PBT matrix molecules shows the phenomenon of self-inside-out splitting, which indicates that the stress distribution in the interface region changes in the case of larger defect widths, and the influence of defects on the mechanical behavior is more obvious. This transition in strain response may be related to the increased interfacial interaction area, causing external forces to concentrate more on the bulk matrix region. In summary, the variation in defect width significantly influences the strain behavior of the PBT matrix molecules during the tensile process. Under smaller defect widths, the mechanical behavior of the interfacial region dominates, while under larger defect widths, the mechanical behavior of the bulk PBT matrix region becomes the primary influencing factor.

## 4. Conclusions

This study systematically investigated the interfacial mechanical properties and tensile responses of defective and non-defective PBT matrix–AP systems under uniaxial tensile deformation using molecular dynamics (MD) simulations. The effects of temperature, cross-linking degree, tensile strain rate, and AP defect size were analyzed. The key findings are summarized as follows:

(1) At low temperatures (e.g., 200 K), the PBT–AP interface exhibited high stress peaks and brittleness characteristics due to restricted molecular chain mobility. In contrast, when at low temperatures (e.g., 500 K), the molecular chain dynamics increased, the stress peak decreased, the plastic flow was prolonged, and there was a transition to a viscoelastic dominated ductile deformation.
(2) The interfacial adhesion strength and tensile resistance of the PBT matrix were strongly influenced by cross-linking density. A 90% cross-linking degree formed a robust molecular network with strong intermolecular interactions, yielding the highest stress peak (39 MPa) and optimal resistance to interfacial debonding. In contrast, a 70% cross-linking degree resulted in rapid post-peak stress decay due to a loosely connected network, while an 80% cross-linking degree exhibited delayed stress accumulation due to inefficient interchain stress transfer.
(3) Higher strain rates (5 × 10^10^ s^−1^) increased peak stress and reduced deformation time, as molecular chains had limited time for relaxation and rearrangement. Lower strain rates (5 × 10^9^ s^−1^) facilitated gradual slippage and reorientation of molecular chains, enabling more uniform deformation and higher residual stress retention.
(4) Defect size significantly influenced the interfacial failure mechanism. Smaller defects (20–30 Å) preserved stress distribution patterns similar to those of the defect-free interface, while larger defects (40 Å) disrupted stress transfer, shifting failure from the interface to the PBT matrix and reducing peak stress by approximately 32%.

These findings provide insights into the mechanical behavior of PBT–AP interfaces, which are essential for optimizing the structural integrity and durability of polymeric composites.

## Data Availability

Data will be made available on request.
